# Changing to Concept-Based Curricula: The Process for Nurse Educators

**DOI:** 10.2174/1874434601711010277

**Published:** 2017-12-29

**Authors:** Kristy A. Baron

**Affiliations:** School of Nursing, Weber State University, 3875 Stadium Way Dept. 3903, Ogden, Utah 84408, USA

**Keywords:** Concepts, Concept-Based Curriculum, Nursing Education, Teaching Conceptually, Changing Curriculum, Student-Centered Learning, Teaching-Learning Strategies

## Abstract

**Background:**

The complexity of health care today requires nursing graduates to use effective thinking skills. Many nursing programs are revising curricula to include concept-based learning that encourages problem-solving, effective thinking, and the ability to transfer knowledge to a variety of situations—requiring nurse educators to modify their teaching styles and methods to promote student-centered learning. Changing from teacher-centered learning to student-centered learning requires a major shift in thinking and application.

**Objective:**

The focus of this qualitative study was to understand the process of changing to concept-based curricula for nurse educators who previously taught in traditional curriculum designs.

**Methods:**

The sample included eight educators from two institutions in one Western state using a grounded theory design.

**Results:**

The themes that emerged from participants’ experiences consisted of the overarching concept, *support for change,* and central concept, *finding meaning* in the change. Finding meaning is supported by three main themes*: preparing for the change, teaching in a concept-based curriculum, and understanding the teaching-learning process.*

**Conclusion:**

Changing to a concept-based curriculum required a major shift in thinking and application. Through support, educators discovered meaning to make the change by constructing authentic learning opportunities that mirrored practice, refining the change process, and reinforcing benefits of teaching.

## 1 INTRODUCTION

Complex and changing health care environments require nurses to use effective thinking skills, promoting safety and quality of care for their patients [1, 2]. Many nursing programs are revising curriculum from a traditional educational approach to a concept-based curriculum to improve thinking skills. In a traditional curriculum, the teacher provides extensive content and facts to passive students. Presenting extensive information allows minimal time for students to organize and apply the information and usually results in students memorizing and recalling facts. Conversely, the concept-based curriculum provides selective information for students to organize, apply, and store in their long-term memories for retrieval to new situations [2].

A concept-based design consists of concepts and exemplars or examples [3]. “A concept is an organizing principle, or a classification of information” [4]. Faculty choose concepts that reflect nursing practice and organize the concepts in domains such as biophysical, psychosocial, health care systems, and professional practice [5]. An example of a concept in the biophysical domain is perfusion and is defined as “the flow of blood through arteries and capillaries delivering nutrients and oxygen to cells and removing cellular waste products” [4]. After selection of the concept, faculty choose exemplars or examples to help students understand the concept [2, 5]. The exemplars reflect essential clinical knowledge related to the concept. For instance, potential exemplars for the perfusion concept include heart failure, stroke, and shock. Exemplars provide the way for students to gain a deep understanding of the concept. In-depth understanding of the concept and exemplar support the ability for students to transfer understanding to new situations [2, 4]. Conceptual learning requires the student to link facts and exemplars to concepts through an active process such as completing case studies, questions, or problem-solving activities—encouraging students to practice thinking [2, 5]. Practice thinking in the classroom evolves from a teacher-centered learning to a student-centered learning environment, focusing on clinical practice. However, the development of a concept-based curriculum does not change the teaching practices of faculty and ways students learn. Subsequently, faculty need to learn ways to present concepts and exemplars in classroom activities that require problem-solving, and students need to understand how to connect facts and concepts with exemplars for in-depth understanding [6]. However, faculty may voice concerns about the change, such as revising curriculum requires time in their already busy schedules, not recognizing the benefits of implementing a concept-based curriculum, and feeling unprepared in designing curricular and instructional designs. Minimal information exists concerning faculty concerns and potential barriers to implementing a concept-based curriculum [5, 7]. Therefore, the following research study explores the perceptions of nurse educators who experienced the process of changing to concept-based curricula, including their insights of teaching practices and their views of the teaching-learning method [8].

### 1.1 Understanding the Level Of Thinking for Future Nurses

For over a decade, nursing education has struggled to meet workforce expectations; subsequently, calls for curriculum reform have been issued [9-12]. Responding to calls for reform has resulted in many educators revising their curriculum from traditional designs to concept-based curricula, as one method to lessen the gap between practice and education [13, 14]. Traditional curricula is defined as teacher-centered learning and focuses on memorizing facts and information, transferring large amounts of information to passive students, and limiting time to problem-solve in the classroom. Comparatively, concept-based curricula is described as learner-centered and focuses on understanding concepts using exemplars, facilitating active learning by requiring students to apply concepts in clinical contexts, and allowing students to transfer concepts in unfamiliar contexts: To practice thinking [15-17]. Two landmark reports support the processes of a concept-based curriculum.

The report, *The Future of Nursing: Leading Change, Advancing Health*, recommended that nursing education transform from current teaching methods rather than adding more information into a saturated curriculum. In addition, nursing education was to focus on teaching fundamental concepts at different levels of learning in various contexts rather than using the traditional method of rote memorization [10].

The second report supports the current transformation of nursing education to meet the demands of health care, *Educating Nurses: A Call for Radical Transformation* [18]. Due to the findings of the study, four essential changes were suggested to help nursing programs reduce the gap between practice and education: first, focus on concrete concepts in context and thinking that can be applied to practice; second, extend practice to the classroom; third, expand critical thinking to include multiple ways to think; and fourth, include intentional methods to internalize ways to develop professionally in nursing.

Because teaching in the classroom traditionally consists of transmitting large amounts of information to passive students with minimal time to problem-solve, the practice of thinking in the classroom is limited. Nurse educators are looking for ways to promote thinking in the classroom while using problem-solving skills; consequently, many nursing programs are changing to a concept-based curriculum [14]. The processes of changing to a concept-based curriculum include designing conceptual curricular structures, teaching conceptually, and evaluating teaching–learning methods through a conceptual lens [6].

### 1.2 Designing Conceptual Curricular Structures

Managing large amounts of available information continues to be an issue. One way to do so is by using concepts. A concept, or set of concepts, organizes information; therefore, it is easier to understand and remember information compared to a list of facts [3]. Exemplars are used to provide specific contexts for a concept, helping students understand the concept more thoroughly [2]. The type of exemplar used can increase the level of learning. For instance, the exemplars, adult respiratory distress syndrome and pneumonia, can be used for the gas exchange concept [3]. However, caring for the patient with adult respiratory distress syndrome will require a higher level of knowledge and skill compared to caring for a patient with an uncomplicated pneumonia.

### 1.3 Teaching Conceptually

The concept-based teaching approach employed in concept-based curricula has existed for more than 50 years and focused on the development of complex thinking. Initially, this method was developed by Hilda Taba and was popular during the 1960s and early 1970s in the United States. This corresponded to the implementation of the *open classroom* in kindergarten (K) to 8^th^ grades, which reflected team teaching, creativity, and less structured curricula. However, an educational movement in the mid-1970s changed the dynamics of the open classroom. The purpose of this later movement was to promote evaluation of students more accurately using objective tests. These tests focused on segments of information and skills but were incapable of measuring complex thinking skills. The focus of teaching and learning changed, yet again, to meet the criteria of objective tests, and the concept-based method with the emphasis on teaching thinking waned, but the method has resurfaced in nursing programs and in other disciplines today [16, 19].

Erickson’s work instrumentally demonstrated the benefits of a concept-based curriculum and instruction for the 21^st^ century. Her work was based on the findings of Hilda Taba. Erickson described the task of developing curricula and teaching students to think. She stated, “Designing curricula to develop thinking is hard work; teaching students to think is even harder work” [16].

Similar to pilots, the profession of nursing requires learning knowledge and skills. Benner’s model of skill acquisition and application of knowledge in nursing, Novice to Expert, is based on the Dreyfus Model of Skill Acquisition that was developed through studying different types of groups, such as chess players and Air Force pilots [20, 21]. This method was based on performances in situations and learning through experiences. The acquisition of skills and the application of knowledge require the practice of thinking in the context of specific situations. Students need a practice environment to support their practice of thinking and performing skills: Active learning activities in the classroom. Active learning activities that are student-centered need to reflect real life experiences happening in health care today. Benner **et al**. instructed nurse educators to extent practice to the classroom, preparing students for the health care workforce [18]. Extending the practice environment to the classroom is key. This action can be accomplished through implementing concepts and exemplars in case studies, problem-solving activities, and simulations [2, 18]. The purpose for active learning activities is to bring the students as close to the real experience as possible in the classroom, which takes time and creativity. Because active learning in the classroom takes time, students need to be prepared before class to practice thinking in the classroom. One way to support student preparation for active learning in the classroom is through *flipping the classroom* [22].

The viewpoint supporting the method of flipping the classroom means students come to class prepared and ready to practice thinking. The preparation may include PowerPoints with voice, video lectures, readings, and quizzes. When students are prepared for class, they are ready to apply concepts and exemplars into real life situations reflective of current practice in health care. The classroom environment becomes interactive with the educator facilitating learning [22].

### 1.4 Evaluating the Teaching–Learning Methods Through a Conceptual Lens

Nearly twenty years ago, higher education began to shift from an *instruction paradigm* to a *learning paradigm*. The reason for this shift was that educators recognized that the goal or outcome of higher education should be to produce learning, not provide instruction. The end (learning) should guide the means or the way to get there, not the other way around, with the means (teaching) guiding the end. During this time, most of the focus was on the instruction paradigm, and a shift was slowly moving towards a learning paradigm [15].

The instruction paradigm focuses on teaching compared to the learning paradigm, which is about fostering learning. In the instruction paradigm, emphasis is placed on delivering knowledge to passive learners who recall information for a test: The teacher controls the learning. An example of the instruction paradigm is typically described as a lecture format and defines the teacher’s job as covering information. This type of teaching is expected to meet the needs of all learners [15].

In contrast, the learning paradigm involves students discovering and constructing knowledge, which requires active thinking. Constructing knowledge consists of creating frameworks of concepts and skills that can be applied in different situations. This prepares students with a solid foundation to understand and act and to be able to solve problems in various contexts. Activities are learner-centered and address the idea that students learn in various ways and at different rates. Moreover, the learning paradigm supports any learning method that is successful in meeting outcomes [23, 24]. The teacher is the designer of learning and learning methods are continuously restructured to foster learning. The learning paradigm supports collaborative learning with the teacher described as a coach or facilitator of student learning and focuses on education for understanding and learning in context [15, 24, 25]. Both the educator and student take responsibility for the students to learn and achieve learning outcomes [2, 15]. Learning is about change and taking responsibility and consists of taking action by changing behavior in order to meet outcomes [25].

According to Barr and Tagg [15], it will take decades before teachers fully apply the learning paradigm and the gradual process will require experimentation for ultimate success. However, trying to apply a part of the learning paradigm to the instruction paradigm is ineffective. In order for the students to become proficient in thinking, they need opportunities to think. Changing to the learning paradigm will require action, hard work on the part of the teacher [15]. Concept-based curriculum and teaching conceptually support the learning paradigm.

Because there is limited time in the classroom, students become responsible for their learning. Concepts and exemplars can be used to organize students’ learning, helping them transfer learning to other situations: A life-long skill necessary to learn class curriculum and to succeed in their workplace when new problems are encountered [4, 24, 25].

The purpose of this qualitative study was to explore the processes of eight nurse educators changing from a traditional curriculum to concept-based. The study included three specific aims: analyze nurse educators’ perceptions and behaviors related to changing from traditional to concept-based curricula; explore the teaching practices of nurse educators who have changed to a concept-based curriculum; and examine how nurse educators perceived the teaching-learning process in concept-based curricula [2].

## 2 METHODS

### 2.1 Participants and Setting

The study used a purposive sample of eight nurse educators who had recently transitioned from traditional to concept-based curricula. Inclusion criteria for the participants coincided with the National League for Nursing (NLN) eligibility requirements for the Certified Nurse Educator Exam [26]. Other criteria for participants included the following: experienced teaching in a concept-based curriculum for a minimum of two consecutive semesters, employed full time in a higher education institution, and reflected current assignment of teaching face-to-face and/or online classes. The researcher identified nursing programs that adopted the concept-based curriculum through textbook vendors. After programs were identified, the researcher approached and invited an educator at each university to participate in the study. The educator at each university then recommended other colleagues who met criteria and showed interest in participating in the study. All the interviews were conducted at the participants’ institutions of employment.

Educators from two types of nursing programs were selected from two universities: registered nurse to baccalaureate and prelicensure baccalaureate. Although the programs’ curricula differed, the focus of the grounded study examined the process for nurse educators changing from a traditional curricular design to concept-based curriculum.

### 2.2 Ethical Considerations

Approval from Institutional Review Boards from two universities in one Western state was granted. Written informed consent was provided for participants. To ensure subject protection, names were changed to conceal participants’ identities, and participants were guaranteed that their names would remain confidential in any data from the study. In addition, participants had the right to withdraw from the study at any time. Furthermore, data from participants remain in the researcher’s locked safe.

### 2.3 Data Collection and Analysis

The data was collected and analyzed by one researcher. The researcher conducted semi-structured interviews with key questions and question prompts. Questions were open-ended and constructed in a way to allow the participants to choose their direction for a response. Length of interview included 60 to 90 minutes per interview. The researcher used a digital device to record each interview, and interviews were transcribed by professional transcriptionists. Interviews generated 130 pages of information; subsequently, the software program, NVivo 10®, was used to help manage and analyze data.

Limited nursing literature exists regarding the processes of implementing a concept-based curriculum; therefore, grounded theory was used in the study, reflecting Charmaz’s methods [5, 27]. The methods used finding the action in each segment or line. Finding action line by line was a process that helped to focus on analysis and prevent concepts and theories from emerging too soon and risking errors. During this initial phase, gaps were identified; therefore, this phase provided direction to obtain future information. The aim was for the codes to fit the data, not for the data to fit the codes. While coding data, it was necessary to constantly compare the data as each new interview was analyzed [27]. This method was inductive using the process of constant comparative methods, and with current data determining what additional data was sought. The second phase of coding, focused coding, identified the most significant and frequent codes and provided criteria to review the data. The purpose of this process was to evaluate the strength of these codes in order to categorize the data. Frequent and significant codes were categorized and identified. Codes with similar meaning were merged. The goal of focused coding or the second cycle of coding was to develop thematic organization [27]. The third phase, axial coding, organized previous codes and categorized information into subthemes and experiences, with the subthemes being supported by the experiences. While completing focused coding in the NVivo 10® program, subthemes and experiences emerged along with links that represented a type of axial coding [27]. The fourth phase, theoretical coding, illuminated relationships among the subthemes. Theoretical codes identified possible relationships among the subthemes; therefore, themes emerged into a theoretical direction [27]. The NVivo 10® program allowed a visual representation of the coding words and displayed the associations with each code and the number of times the code was referenced. Memos were used by the researcher to record reflective thoughts [27]. Data represented faculty experiences, as a whole. Selection of participants along with gathering of data continued until no new information emerged or saturation of the data was reached [27].

### 2.4 Standards of Rigor for the Study

Standards of rigor for this qualitative study included credibility, transferability, dependability, and confirmability. *Credibility* was supported by having participants examine their transcriptions, which allowed for opportunities of clarifications. Furthermore, experts in grounded qualitative research compared data with themes and agreed with findings. *Transferability* was supported through details of the settings and characteristics of the sample. *Dependability* was demonstrated by findings being consistent with data and confirmed through an audit trail. Techniques used to establish *confirmability* included audit trail, triangulation, and reflexivity [28, 29].

## 3 RESULTS

Eight participants selected shared experiences that formed the data: interviews, documents of lesson plans, and demographic questionnaires. Demographics showed participants’ age ranges include 31 to 62 years with a mean age of 48.3 ±11.75 years, practice as a clinical nurse spanned 20 ± 10.68 years, and teaching as an educator averaged 12.38 ± 6.44 years. All participants were female—seven Euro-American and one Dominican. The education of participants included four master’s degrees in nursing—two currently enrolled in doctoral programs, two doctorate degrees, one nurse practitioner enrolled in a doctor of nursing practice (DNP) program, and one DNP degree [8].

For both programs, implementing a concept-based curriculum was not a gradual process. One program had revised all of their courses from a traditional to a conceptual curricular design over a two-year period; whereby, the other program revised their courses over a one-year period. Prior to the study, one nursing program had implemented their concept-based curriculum for two semesters compared to the other program that had implemented the curriculum for approximately eight semesters. Participants who taught the concept-based program longer shared more information about evaluative planning; therefore, these participants were represented to a greater degree when discussing evaluation.

From the participants’ experiences, 18 subthemes emerged and were organized into three themes corresponding to the aims of the study: Preparing for the Change (5 subthemes), Teaching in a Concept-Based Curriculum (7 subthemes), and Understanding the Teaching-Learning Process (6 subthemes). These themes were supported by participants’ experiences. Participants indicated through their experiences ways they changed to implement a concept-based curriculum. An overarching concept includes *support for change*. Support is required to effectively find meaning or understand the processes of a concept-based curriculum, and support was needed to prepare for the change, teach in a concept-based curriculum, and understand the teaching-learning process. Through support, participants were able to understand or to *find meaning* and construct learning to reflect student-centered approaches, foster authenticity by offering real experiences in class, refine change through evaluative methods, and reinforce benefits of teaching.

### 3.1 Preparing for the Change

The theme, *Preparing for the Change*, consisted of five subthemes: driving factors for change, planning the change, changing our thinking, supporting the change, and recognizing challenges. The first subtheme, driving factors for change, included meeting the needs of the nursing program by updating the curriculum and solving content overload. Julie explained, “Well, we knew that it [the curriculum] needed [to be] updated. . . . We knew that was going to be a major overhaul and it was going to be a huge learning curve for the faculty, but nevertheless, we went that direction.” Lauren discussed, “I think the big thing was that there were just too many diseases, too many pathologic processes, that it became so overwhelming for students.”

Second subtheme, planning the change, showed most of the participants served on committees while revising the curriculum. Planning included developing concepts and exemplars while incorporating lifespan development. Julie was on the initial committee that began the change at her institution, and she shared, “I sat on the revised curriculum committee, which was a group of us with community representation that decided how this was all going to work and that was planning the entire process of the curriculum change: from teaching the faculty the new system to setting up the committees that would work on it, determining what the philosophy was and [what] the School of Nursing competencies were, and choosing our philosophical stance for that, as well as kind of a conceptual model to go from. So, that kind of encompassed everything, and then I became part of different committees.”

Third subtheme, changing our thinking, showed ways participants’ thinking had changed. Participants shared they learned to let go of content and teaching methods. For example, Audrey stated, “I think less about teaching all of the details.” Similarly, Sarah tailored her maternal-newborn class to a nurse that may experience a pregnant patient in a setting besides labor and delivery. She stated, “What is it they absolutely must know.”

Fourth subtheme, supporting the change to a concept-based curriculum required faculty to communicate and become educated about processes of a concept-based curriculum. Sarah stated, “We had it all so neatly planned out, at least on paper, but then as it gets into reality it gets messy and changes things. I think that to do it well it really does take a great deal of coordination and time on the [part of the] faculty, talking with each other about what they are doing.” Besides communication, educating faculty was a main component in supporting the change to the concept-based curriculum and included meeting with experts, reading literature, attending workshops, and self-teaching. These different methods of education helped to educate participants who were at different levels of understanding the processes of a concept-based curriculum.

Fifth subtheme, recognizing challenges, participants’ comments included the following: limited time to complete assignments, increased workload, lack of understanding of curriculum design, and lack of experience teaching lifespan development. Sarah summarized the experience succinctly, “We rushed and we had to redo things when we rushed and it was painful.” Lauren confirmed that faculty were rushed to meet deadlines; consequently, they did not get enough input from others. A challenge that many participants expressed was the lack of understanding of curriculum development and specifically a concept-based curriculum. Julie voiced, “One of the big challenges I saw was a lot of the faculty did not understand curriculum development.” Rhonda discussed the struggles her faculty were experiencing: understanding the difference between a concept and content. Lauren noted that some faculty members thought they could transition the content of their courses into the concept-based curriculum. “A lot of people thought, ‘I can just transition my old stuff’ and [they] actually found that it did not work. I basically had to redo all of my courses that I had done, even clinical.” In addition, she described how faculty had difficulty integrating the lifespan in their course because they did not feel comfortable teaching pediatrics or gerontology. Mary expressed the difficulty of changing to a concept-based curriculum and the refining that is required after the change: “It is not going to be perfect the first time you implement it.”

### 3.2 Teaching in a Concept-Based Curriculum

Participants shared experiences of teaching in a concept-based curriculum, and seven subthemes emerged as follows: choosing concepts, preparing for class, getting students to participate, teaching conceptually, describing student-centered learning, changing to andragogy (teaching adult learners), and comparing online to face-to-face.

First subtheme, choosing concepts, participants described a concept to mean a common thread or overarching word(s) describing content. Rhonda shared, “Many people are so tied to their content that they can’t see that they could teach that content while under the umbrella of the concept itself.” Participants located concepts by various means and resources such as published texts, articles, the Baccalaureate Essentials document, and National Council Licensure Examination (NCLEX) blueprint.

Second and third subthemes, focused on preparing for class and getting students to participate. Rhonda shared how teaching in a concept-based curriculum focuses on learning, and instruction is based on student participation; consequently, students need to be prepared for class. Rhonda stated, “It is a struggle and sometimes they don’t come prepared and then we have a discussion about what we are doing here. In the past, she has stated to her students, “You are not prepared. Go home.” A component of conceptual teaching is student-centered learning. This means students become actively involved in learning activities; therefore, it is important to get students to participate in class by giving them a safe environment. Julie shared this can be accomplished by beginning class with an activity that encourages participation and teamwork and creates a more relaxed atmosphere.

Fourth subtheme, teaching conceptually, several participants interpreted the meaning of teaching and learning concepts. Mary discussed, “It means that . . . the student is able to transfer knowledge from the concept to any situation.” Lauren agreed about transferring conceptual knowledge and described how she taught conceptually: “You would have exemplars and you would have focal points. . . I could teach that principle [concept] then students could apply . . . if I gave them an asthma care scenario or a chronic obstructive pulmonary disease case.” Rhonda shared methods of teaching conceptually by connecting students to real-life experiences, guiding learning through questions, and allowing students opportunities to solve problems by using appropriate nursing interventions. Denise felt that she was somewhat teaching conceptual before, but expressed how concepts streamlined her teaching to stay on track.

Fifth subtheme, describing student-centered learning, most participants described student-centered learning as students being self-directed, participating actively, and learning through actual experiences or simulations while the instructor facilitated learning. In a maternal-child class, Sarah required her students to review the voice recordings with PowerPoints before attending class; thereby, the students were prepared in class to think through case studies and questions. Lauren taught a hybrid medical-surgical course with each week consisting of two hours online before class and three hours in class. The online portion consisted of activities to prepare students for class such as voice recordings with PowerPoints, pre-readings that covered concepts with pathophysiology, and graded quizzes to assess their learning; consequently, students were prepared for class that allowed instructors to focus on nursing management and priorities. In class, case studies were organized in slides requiring student input. These same case studies were included on the exams.

The last subthemes, changing to andragogy and comparing online and face-to-face teaching were mentioned by some participants. Regarding andragogy, Rhonda described how she needs to be prepared for class, “I can’t just pull my lecture out and pull out my PowerPoint. That doesn’t work anymore. I can’t be unprepared because we won’t have anything to talk about.” Some felt that concepts lose strength online: Audrey stated, “But still, I have a big concern about making the online classes I teach more alive.”

### 3.3 Understanding the Teaching-Learning Process

The theme, Understanding the Teaching-Learning Process, was supported by six subthemes that emerged from participants’ experiences: noticing benefits of teaching, expressing enjoyment about teaching, bridging academia and practice, recognizing students’ wants and needs, influencing students to change their thinking, and teaching effectively.

The first two subthemes of noticing benefits of teaching and expressing enjoyment about teaching revealed participants enjoyed helping students to learn and found satisfaction in interacting with students. Third subtheme, bridging academia and practice, Lauren shared the following: “We discuss a concept in clinical, they also discuss that same concept that week in class—so they are aligned.” In addition, Julie compared memorizing facts and understanding concepts while working with new nurses in the intensive care unit setting. The new nurses had lists of memorized facts to try and trouble shoot problems instead of understanding basic concepts of perfusion such as CO=HRxSV. Julie confirmed that lists “did not work for the real world.” Fourth subtheme, recognizing students’ wants and needs, Denise shared one obstacle and advantage regarding a policy course, “They do not understand the value of it for them from a clinical perspective sometimes. . . . The whole expansion of knowledge sharing and ability to have even novice nurses have expertise and a lot of insight on a particular concept that perhaps I had not thought of or most times their student peers had not thought of; I think that is satisfying.”

Fifth subtheme, influencing students to change their thinking, Mary observed that students were still compartmentalizing their thinking because of the way a concept-based curriculum is taught—“teaching the concept of perfusion and you are really just teaching cardiac.” Mary said we should be able to bring up any disease and examine ways it influences perfusion. Sixth subtheme, teaching effectively, participants stated that they allowed student opportunities to think by connecting students to real-life experiences.

## 4 DISCUSSION

### 4.1 Implementation of Change Model and Continuous Education for Faculty

Brady **et al**. [6] discuss the challenges of implementing a curriculum change and successful ways to implement a change model. Over time, faculty rely on the same teaching practices and become attached to particular subjects. Consequently, introducing curriculum changes can cause faculty to feel frustrated, meeting change with resistance [5]. In addition, in concept-based curriculum, typically, traditional courses such as adult health, maternal-child health, and mental health, might be combined into one course [14]. Faculty may express concerns about teaching across the lifespan and lacking expertise in specialty areas.

Revising curriculum requires well-planned approaches along with effective change strategies for successful change [6]. In addition, becoming adept in this teacher-learner role in the active learning environment requires a change in thinking and teaching skills on the part of the educator. Since this necessarily constitutes a change in thinking and behavior, review of a change model is helpful in understanding this process.

Faculty can easily underestimate this process of change; subsequently, a model can guide faculty through the complexity of change [6]. A change model, such as Kotter’s Eight-Step Change Model focuses on helping people accept and become prepared in their work culture: establishing a sense of urgency, creating the guiding coalition, developing a vision and strategy, communicating the change vision, empowering broad-based action, generating short-term wins, consolidating gains and producing more change, and anchoring new approaches in the culture [30]. Sense of urgency helps faculty understand the importance and benefit of the change. The guiding coalition represents a team with strong leadership and experts of conceptual curricular designs, conceptual teaching, and evaluative teaching-learning methods. The vision and strategy guide the change process. Furthermore, thorough and consistent communication promotes the vision and strategy. Empowering broad-based actions focuses on reducing barriers to support the change. Generating short-term wins promotes recognition of improvements. Consolidating gains and producing more change continue the momentum to secure the change throughout the program. Finally, anchoring new approaches, such as concept-based curriculum, becomes the standard for the program [30].

Both groups of participants agreed the major driving force for changing to a concept-based curriculum centered on solving the problem of content overload in their curricula. While making the change to concept-based curriculum, educators shared similar barriers such as lack of time, which excluded input from others. In addition, increased workload caused personal and workplace stress for participants. Participants shared they participated actively in the change process by helping faculty move from traditional ways of transmitting information through lectures to focusing on student-centered activities reflective of concepts and exemplars. Content in their courses changed from large amounts of information to selected concepts and exemplars, reflecting what students must know to perform safely as a generalist nurse. Participants needed to learn how to design conceptual curricular structures, implement conceptual teaching strategies, and evaluate teaching-learning methods through a conceptual lens. An observation included when faculty tried to move their content-laden course into a concept-based course, they discovered the transition proved ineffective.

 However, participants shared that faculty members showed different levels of understanding in curriculum designs, teaching strategies, and teaching-learning evaluative processes. Participants shared experiences about mentoring other faculty members and becoming frustrated with faculty who did not take the time to understand components of conceptual curricular designs. Because faculty members remain at varied levels of understanding of concept-based curricular designs, conceptual teaching, and teaching-learning evaluative methods, education needs to be ongoing, such as meeting with experts, reading literature, attending conferences and workshops, and receiving peer tutoring.

### 4.2 Requirement of Concept-Based Curriculum: Conceptual Teaching

Besides developing curricular designs, conceptual teaching is a skill that requires time and effort to learn. Brady **et al**. [6] hired consultants outside of their program, both nursing and non-nursing disciplines, to educate their faculty on conceptual teaching. Conceptual teaching requires specific components: concepts and exemplars. Participants located concepts by various means and resources. Currently, a benchmark approach for choosing concepts remains available in literature to support educators in this effort [4].

Teaching conceptually includes a learner-centered approach with active learning occurring in the classroom. The change can be challenging when a teacher is changing from a teacher-centered approach and focusing on content [6]. Participants shared that student preparation remained essential to promote participation in student-learning activities in class. During class, participants facilitated learning through supplying concepts and exemplars, guiding the learning process. They expected students to apply concepts and understanding in other situations—transfer learning. Activities in class reflected problem-solving and thinking exercises. Participants shared preparing for class takes time and creativity. Class time is not a matter of providing a lecture through PowerPoint, rather bringing the students as close as possible to actual clinical experiences through well-developed activities.

### 4.3 Successful Teaching-Learning Process Through Transferring Understandings to Various Situations

Conceptual teaching helps learners organize information in the long-term memory of their brain for transferring and applying information to new situations. Conversely, when facts are presented, usually they are unconnected and pose a problem for the learner to remember and retrieve for transferring to new situations. According to Giddens, Caputi, and Rodgers [2], “a conceptual organization of information is foundational to clinical reasoning.” Key to conceptual learning remains the deep understanding that occurs with exploring concepts with exemplars that reflect complex cases reflective of current practice. With deep learning, learners notice patterns and relationships that can be transferred to new situations [2]. The classroom becomes an effective environment to transfer deep understandings to new situations—practice thinking.

Participants shared that organization of curriculum showed concepts aligning in didactic and clinical settings. In addition, participants realized problem-solving from memorized lists remains impossible; however, effective problem-solving happens through understanding concepts and exemplars. Furthermore, adult learners bring insights and experiences that differ from others and enrich the learning environment. For nursing-student learners, instead of recognizing the diagnosis of a patient, such as heart failure, students focus on the concept of perfusion and complex issues that surround the concept. A challenge remains to expand students’ ways of thinking through using a concept with various exemplars. Participants shared the importance of extending practice to the classroom by presenting real life experiences through case studies, simulations, and problem-solving activities.

### 4.4 Study Limitations and Recommendations

The data from the study was limited to registered nurse to baccalaureate and prelicensure baccalaureate programs. Delivery of the courses varied such as face-to-face, online, and hybrid (face-to-face and online) formats. In addition, the length of time each program had implemented the concept-based curriculum differed between the two settings: two semesters compared to eight semesters. The exploration of the topic, changing to a concept-based curriculum, was restricted to questions concerning the didactic portion of the curriculum. Recommendations for future studies include assessing students’ perceptions regarding concept-based curriculum, implementing teams to support lifespan learning, and comparing teaching conceptually in clinical and didactic settings, including online, hybrid, face-to-face courses.

## CONCLUSION

Educators require sufficient support when revising curriculum from a traditional approach to concept-based. Finding meaning or understanding the processes of a concept-based curriculum helps educators refine curricular structures, teaching techniques, and teaching-learning evaluative methods. Concept-based teaching requires educators to construct learning using concepts and exemplars while implementing student-centered learning activities with authentic experiences from clinical practice. Deep learning can occur with authentic experiences with the ability for students to transfer understandings to new and various situations in preparation for a complex and changing health care environment.
